# A Preliminary Study on the Effect of Macro Cavities Formation on Properties of Carbon Nanotube Bucky-Paper Composites

**DOI:** 10.3390/ma4030553

**Published:** 2011-03-10

**Authors:** Ludovic Dumée, Kallista Sears, Jürg Schütz, Niall Finn, Mikel Duke, Stephen Gray

**Affiliations:** 1 CSIRO Materials Science and Engineering, Bayview Avenue, Clayton 3168, Victoria, Australia; E-Mails: kallista.sears@csiro.au (K.S.); jurg.schutz@csiro.au (J.S.); niall.finn@csiro.au (N.F.); 2 Institute for Sustainability and Innovation, Victoria University, Melbourne VIC 8001, Australia; E-Mails: mikel.duke@vu.edu.au (M.D.); stephen.gray@vu.edu.au (S.G.)

**Keywords:** carbon nanotube, bucky-paper, sacrificial beads, tuning porosity

## Abstract

In this study, we focus on processing and characterizing composite material structures made of carbon nanotubes (CNTs) and reproducibly engineering macro-pores inside their structure. Highly porous bucky-papers were fabricated from pure carbon nanotubes by dispersing and stabilizing large 1 μm polystyrene beads within a carbon nanotube suspension. The polystyrene beads, homogeneously dispersed across the thickness of the bucky-papers, were then either dissolved or carbonized to generate macro cavities of different shape and properties. The impact of adding these macro cavities on the porosity, specific surface area and Young’s modulus was investigated and some benefits of the macro cavities will be demonstrated.

## 1. Introduction

Carbon nanotube (CNT) composite materials have been investigated since their discovery. CNTs exhibit exceptional electrical, thermal and mechanical properties [1]. Over the last decade they have attracted a lot of interest and efforts were made to incorporate them efficiently into composite material structures. CNTs can be easily processed as bucky-papers (BP), which are entangled meshes of nanotubes. BPs have been studied and their properties investigated [2,3,4]. They exhibit promising properties while being strong and flexible structures to engineer. Intrinsic properties of CNT BPs, such as their high hydrophobicity [5] and their very high natural porosity [6], should be exploited to produce high performance composites. Furthermore, CNT BP’s surfaces [7] can be easily functionalized or combined in composite structures to emphasize their properties. Very thin and robust self-supporting BPs can be reproducibly fabricated from well dispersed CNTs [3]. Recent work has shown that they can be processed in large and thick 3-D structures [8,9,10] offering very interesting elastic mechanical behaviors. Furthermore, it was also shown that after being tested in compression their initial geometries and dimensions could be nearly fully restored by immersing them in an appropriate solvent [11]. Tuning their pore size distribution [12] or surface porosity by dissolving poly(styrene) spheres was reported [13] and showed high promise in engineering macro-structured membranes but issues related to pore cloaking and to reduced diffusion were however raised. Previous work involved testing pure self-supporting CNT BP membranes as well as composite material CNT based membranes [14,15]. It was shown that those membranes had specific surface areas up to 200 m^2^ g^−1^ and porosities reaching 90%, while having Young’s Moduli close to 1 GPa in tensile tests. BPs can be templated with polymers [15] or metals [16] to improve their mechanical or transport properties but little work has been done to effectively test the change in porosity on the permeation properties.

Controlling porosity and surface area is of prime importance to engineering structures for improved separation performance with specific surface properties. In this study, composite poly(styrene) beads/BPs were fabricated from pure CNTs and templated with 1 μm polystyrene sacrificial beads. The polystyrene beads, homogeneously dispersed across the thickness of the BPs, were then either dissolved or carbonized to generate macro cavities of different shape and properties. The impact of adding these macro cavities on the porosity, specific surface area and Young’s modulus was investigated and some benefits of the macro cavities will be demonstrated.

## 2. Experimental Section

### 2.1. Processing of the CNTs Based Composite Materials

Propan-2-ol/CNT 20 mg/L suspensions were produced by sonicating chemical vapor deposition grown CNTs [17] at 150 W and for sequences of 15 min. The samples were processed by uniformly mixing 1 μm polystyrene (PS) latex beads (purchased by Sigma Aldrich, reference L2157) with the solvent dispersed CNTs at a ratio of 0.05 mg bead/L. The mixed suspensions were filtered through a Millipore filtration unit to obtain a self-supporting polystyrene/CNT BP following the procedure in [6]. A 200 nm pore size poly(ether-sulfone) membrane was used as a support for filtration and was removed by peeling-off after drying. Permanent low speed stirring was applied to the solutions to avoid decantation and phase separation. A first series of samples was backwashed and dipped for 24 hours at room temperature in technical grade dimethyl-formamide (DMF) to dissolve the polystyrene based-latex beads, while a second series of samples was heated in a 99.9% pure N_2_ atmosphere at 350 °C to carbonize the polystyrene beads.

### 2.2. Characterization Techniques

Scanning Electron Micrographs (SEM) of sample cross sections were obtained by Focus Ion Beam (FIB) milling the surface of the BPs with a Gallium ion beam. An average BET surface area was determined by N2 adsorption on a Micromeretics Tristar 3000 [18]. The samples were first degassed for 70 hours at 120 °C and then analyzed at 77 K. Pure CNT BPs were used as references and compared with the composite samples. Bubble point [19,20] and pore size distribution were determined by perm-porometry, on a gas flow porometer from Porous Materials Inc., in wet-up/dry-up configuration. A Galwick solution was applied to wet the top surface and the sample pressurized under analytical grade N2. Finally, the mechanical properties of the BPs were evaluated by Instron testing, at 0.5 mm/min at 25 °C and 65% of relative humidity. The samples were cut into 3 to 5 mm wide and 20 mm long strips and tested for their tensile strength on a 10 N load cell. At least 2 samples per series were tested and averaged. The thickness of the samples was also measured at least 5 times with a micrometer prior to undertaking any tests, and the thickness randomly checked on a profilometer Altisurf 500. An average BET surface area was determined by N_2_ adsorption at 77 K on a Micromeretics Tristar 3000 after degassing the samples for 12 hours.

### 2.3. Gas Permeability

Gas permeance measurements were performed by placing the membrane in a 5 cm^2^ o-ring sealed holder which separates a 20 L upstream feed cylinder from a 50 mL permeate cylinder. The feed vessel was isolated from both the vacuum and membrane holder, and filled to 101 kPa with filtered and dry air. For testing, the permeate side was evacuated to a vacuum of 0.6 kPa, isolated from vacuum and the pressure allowed to rise once the isolating valve between the feed and permeate vessels was opened monitored over time until equilibrium was reached. The feed pressure remains essentially constant due to its much larger volume compared to that of the permeate. The membrane permeance, f, can then be determined by transposing the fitting of the pressure rise, P_P_(t), in the permeate vessel with Equation 1. The permeability of the membranes was then calculated by dividing the permeance by the average membrane thickness.

(1) $$P_{P}(t)=P_{F}\left(1-\frac{P_{F}-P_{o}}{P_{F}}e^{\left(\frac{-RTfA}{V}\right)t}\right)$$

where PF is the constant pressure in the feed vessel, P0 is the initial pressure of the permeate, R is the universal gas constant, V is the volume of the permeate, T is the temperature, A is the exposed membrane area, and f (moles m^−2^.s^−1^.Pa^−1^) is membrane permeance.

## 3. Results and Discussion

**Figure 1:**
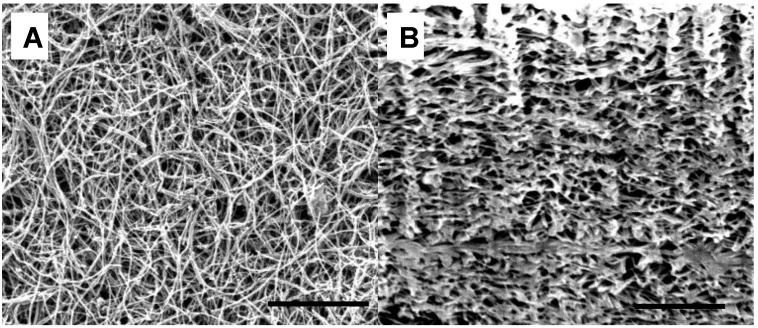
Scanning electron micrographs (SEM) of non bead reinforced bucky-papers (BP). (**1A**) surface; (**1B**) cross section. The scale bar corresponds to 1 μm.

**Figure 2:**
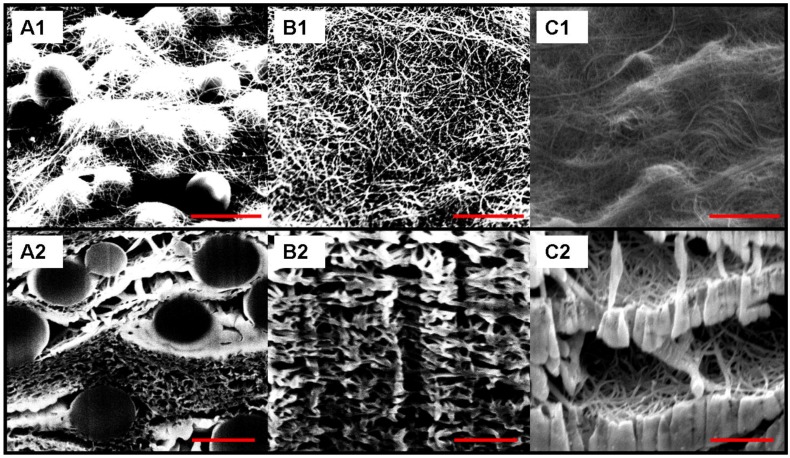
SEM images of BPs surface and cross sections for 1 μm poly-styrene bead reinforced BPs. Images A1 and A2 correspond to the composite after filtration of the bead/CNT suspension, while B1 and B2 were taken on samples after bead dissolution, and C1 and C2 on the bead/CNT composite after carbonization. The micrographs were taken at a tilt of 52°. The scale bars on the micrographs correspond to 1 μm.

As shown in Figure 1A and Figure 1B, Focus Ion Beam milled cross sections of the reference, non templated BPs, the CNT distribution is very even and neither cracks nor macro-bundles were present in the structures (Figure 1). After addition of the beads, the composites were shown to be composed of successions of layers of beads, evenly distributed in small clusters with denser layers of nanotubes (Figure 2 A1 and A2). Furthermore, the presence of the beads greatly changed the global morphology of the BPs, clearly creating macro voids and pockets within the structure as shown after carbonization (Figure 2-C1 and C2). In this case, macro voids were formed in the sites of the beads, thus forming cavities of the diameter of the beads. Additionally, the dissolution of the beads led to the collapse of the cavities, which was attributed to the strong solvent/CNTs interactions and to the PS wicking in such confined spaces (Figure 2 B1 and B2).

**Figure 3:**
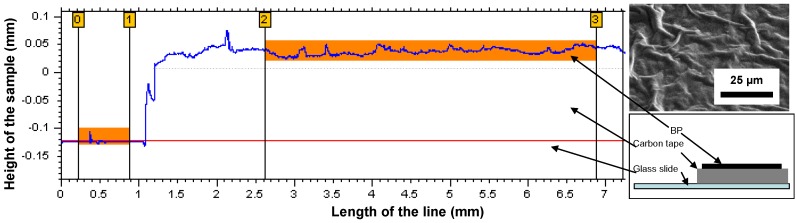
Altisurf measurement of a BP thickness; the sample adhered to carbon tape, and on a glass slide used as a reference. The resolution of the light beam was 20 nm.

The surface roughness and thickness was shown (Figure 3) to vary randomly on the surface according to the presence of macro-bundles in the structure. The surface of the BP was mapped by reconstructing the surface from scan lines as the one presented in Figure 3. Accurately controlling the thickness is critical to processing reproducible composites and to test with a high degree of accuracy their mechanical properties. Average thickness variations were minor and generally limited to a window of 8–10% of the nominal thickness while local larger variations were attributed to CNT macro bundles present on the surface (Figure 3).

**Figure 4:**
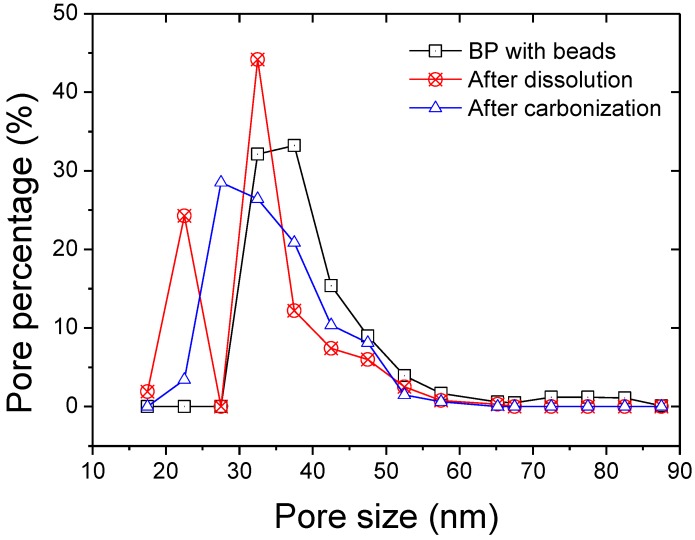
Pore size distribution of the composite membranes determined by perm-porometry.

The pore size distributions of the membranes are given in Figure 4. The distributions tended to narrow after either dissolution or carbonization of the beads compared to the non-treated bead/CNT composite. The average pore size decreased from 37 nm down to 32 and 33, respectively (Table 1).

Furthermore the bubble point of the structures sharply increased after dissolution or carbonization. This was attributed to the tighter pores and also to the reduced porosity. Decreased specific surface area was obtained after both dissolution and carbonization confirming the impact of the beads on the material’s porosity and available surface area (Table 1). This trend was previously reported for infiltrated BPs [15] where PS/CNT composites were found to have specific surface areas up to 70% lower.

**Figure 5:**
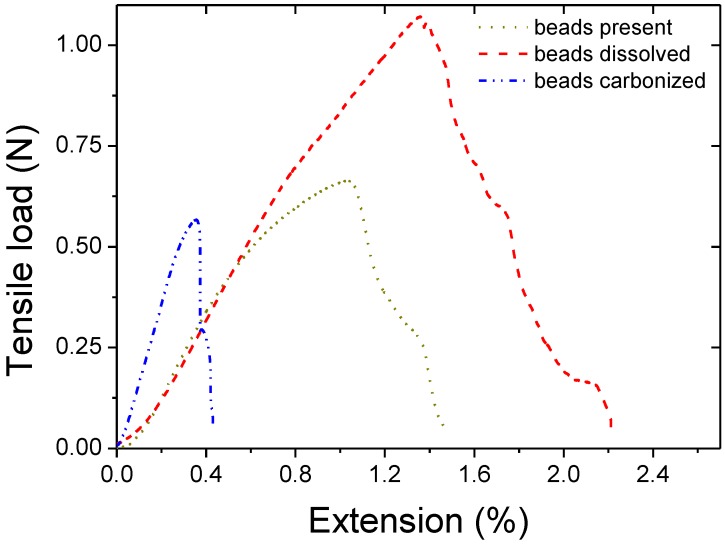
Tensile/extension typical curves for the series of samples.

Typical tensile extension graphs showing the tensile behavior of composite BPs are displayed in Figure 5. The modulus of the structures was found to decrease after simple addition of beads or bead dissolution while it increased after carbonization as reported in Table 1. The decreases of modulus for the two first scenarios were attributed to the defects being generated by the presence of the beads. For the carbonized samples, as shown in Figure 2, shells of carbons were formed at the sites of the beads. The increased modulus was attributed to these carbon shell formations, bridging and linking CNT on a micrometer scale. The shells of carbons literally embedded part of the CNTs within a semi dense matrix which provided higher cohesion and stiffened the composite.

**Figure 6:**
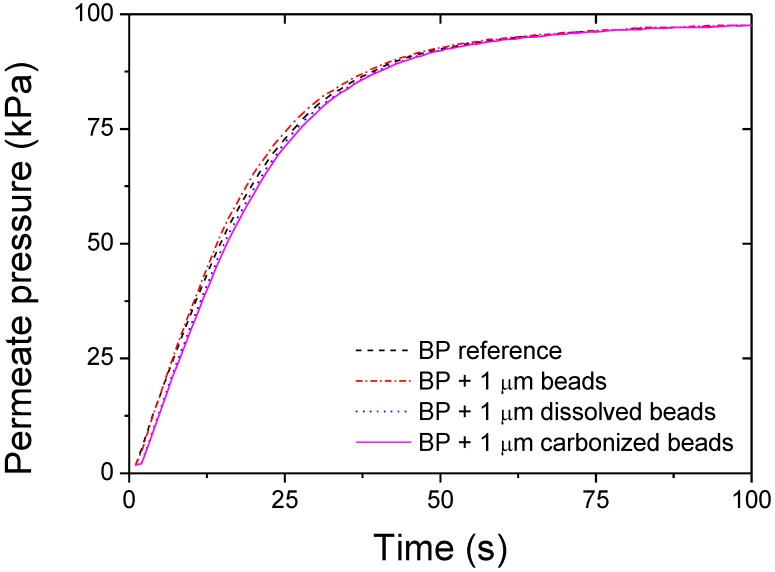
Air permeation across the BP samples.

The air permeation across the porous structures did not seem to be affected by the bead presence (Figure 6). A slight decrease in permeance was found after both dissolution (14.5%) and carbonization (26.8%) compared with the reference sample, which was attributed to the presence of excess organic matter clogging some pores (Table 1). In the case of the dissolved beads, it is likely that the polystyrene was not completely removed from the static dissolution step and led to bridges and aggregates within the BP structure. The polymer presence is confirmed by the FIB SEM cross sections where denser areas can be found (Figure 2). Redeposition of amorphous matter is also visible on the FIB SEMs which correlated with the theory that matter remained present after both bead dissolution and carbonization (Figure 2). Furthermore, after carbonization, the decreased flux can also be attributed to the presence of amorphous carbon being redeposited from the carbonization of the beads.

The BET specific surface was, however, found to be higher after dissolution which is likely due to closed dead end pores at the sites of the beads. Those half closed pores do increase the surface of adsorption but decrease the permeability as no gas can travel across.

**Table 1:** Composite properties.

	Thickness	Tensile Modulus	Average pore size	Bubble point	BET	Permeability
	μm	GPa	nm	kPa	m^2^/g	x10 ^−11^ kg.m^−1^.h^−1^.Pa^−1^
BP reference	10 (+/−2)	0.95	32	783	197	2.61
BP + beads	8 (+/−3)	0.35	37	532	90.7	2.55
BP + beads dissolved	7 (+/−2)	0.21	32	735	135.6	2.23
BP + beads carbonized	6 (+/−3)	0.99	33	760	97.5	1.91

## 4. Conclusions

The use of sacrificial beads and their removal from the BP’s structure by dissolution and carbonization was investigated. After dissolution of the beads no major structural differences were visible although the tensile modulus and bubble point of the BPs were significantly decreased compared to non reinforced BPs. On the other hand, after carbonization, cavities were found in the sites of the beads, thus potentially increasing the BP porosity. Furthermore the carbonized BPs were found to be stiffer than non bead reinforced BPs while otherwise exhibiting similar properties. This increased stiffness was attributed to the amorphous carbons forming bridges between CNTs. This new method to template macro-structures made of nanoparticles was shown to be a promising way to finely control pore size inside BPs even if further work is required to fully foresee the opportunities.

A method to remove the amorphous carbon present in the bead carbonized BP would lead to enhanced permeation membranes and thus is being investigated. Furthermore, the impact of the temperature of dissolution and of carbonization will be investigated as a way to optimize the excess matter removal and better stabilize the structures. Furthermore the use of large grain inorganic salts as sacrificial matter might also be tested to create macro pores. Low propan-2-ol solubility salts could be used to template the BPs with macro particles and be removed with an appropriate solvent in a dissolution step.
